# Assessment in the Supine-To-Stand Task and Functional Health from Youth to Old Age: A Systematic Review

**DOI:** 10.3390/ijerph17165794

**Published:** 2020-08-10

**Authors:** Maria Teresa Cattuzzo, Frederico Santos de Santana, Marisete Peralta Safons, Alessandro Hervaldo Nicolai Ré, Danielle Rene Nesbitt, Ariane Brito Diniz Santos, Anderson Henry Pereira Feitoza, David Franklin Stodden

**Affiliations:** 1 Higher School of Physical Education, University of Pernambuco, Recife 50100-130, PE, Brazil; arianebdiniz@gmail.com (A.B.D.S.); anderson.feitoza.upe@gmail.com (A.H.P.F.); 2 Faculty of Physical Education, University of Brasília, Brasília 70910-900, DF, Brazil; fredericosantosdesantana@gmail.com (F.S.d.S.); mari7ps@gmail.com (M.P.S.); 3 School of Arts, Sciences and Humanities, University of São Paulo, São Paulo 03828-000, SP, Brazil; alehnre@usp.br; 4 Department of Health, Physical and Secondary Education, Fayetteville State University, Fayetteville, NC 28301, USA; dnesbitt@uncfsu.edu; 5 Department of Physical Education, College of Education, University of South Carolina, Columbia, SC 29208, USA; stodden@mailbox.sc.edu

**Keywords:** psychomotor performance, functional evaluation, human development, righting skill, rising from the floor, floor-to-stand, supine rise tasks

## Abstract

Performance in the supine-to-stand (STS) task is an important functional and health marker throughout life, but the evaluation methods and some correlates can impact it. This article aims to examine the studies that assessed the performance of the STS task of young people, adults and the elderly. Evidence of the association between the STS task and body weight status, musculoskeletal fitness and physical activity was investigated, and a general protocol was proposed. MEDLINE/Pubmed and Web of Science databases were accessed for searching studies measuring the STS task directly; identification, objective, design, sample, protocols and results data were extracted; the risk of bias was assessed (PROSPERO CRD42017055693). From 13,155 studies, 37 were included, and all demonstrated a low to moderate risk of bias. The STS task was applied in all world, but the protocols varied across studies, and they lacked detail; robust evidence demonstrating the association between STS task and musculoskeletal fitness was found; there was limited research examining body weight status, physical activity and the STS task performance. In conclusion, the STS task seems to be a universal tool to track motor functional competence and musculoskeletal fitness throughout life for clinical or research purposes.

## 1. Introduction

The human development lifespan perspective provides a framework for studying the changes that occur throughout life [1,2]. Within this perspective, some motor actions are considered developmental milestones and health indicators as the action “rising from a supine position on the floor to an erect standing position” (supine-to-stand, STS), since it is an indicative of bipedal readiness for upright locomotion in children [3] and functional capacity for independence in the elderly [4].

The STS task performance also uncovers an individual’s level of motor competence (MC), defined as the proficiency in motor actions performed with coordination and control [5,6,7]. The STS task requires complex coordination of large muscle groups of the trunk and extremities, while controlling their center of mass in dynamic balance during elevation and stabilizing body alignment during the erect posture [8].

During the 1980s, Ann Vansant proposed developmental sequences for this righting task [1,3,9]. Studies related it to physical fitness and lifestyle variables [10,11,12] expanding the focus of motor development *per se*, to the discussion concerning their relationship with health [6,7]. STS has been investigated at different stages of life [4,8,12,13,14], and unlike other motor skills, such as running or jumping, instruction on how to rise from supine is not taught, which reduces the bias of cultural context or structured practice opportunities. Furthermore, the STS task seems to be a useful marker of health and function problems, predicting serious fall-related injuries [15,16,17].

With these characteristics, maybe the STS task is a useful and practical method to monitor functional MC changes throughout human life and an excellent candidate to be a universal screening tool. However, such assumptions need further examination. For example, health variables as physical activity, musculoskeletal fitness, and body status weight should be considered when evaluating the STS task [14].

Assessing the performance of the STS task or other motor action may involve two different measurement approaches: process and product-oriented. A process-oriented measurement aims to express the quality of the movement, in general, by comparing it with the more successfully mechanical form, as described in the checklists. A product-oriented measurement aims to describe the action results, such as the time to complete a task or the scores on a target, using interval or discrete variables (e.g., seconds, points, speed). Both approaches, complementary, expresses human motor performance [18,19]. Studies using a product-oriented approach measured the STS time by chronometers [20], photoelectric cells [4], or a video [8,12,14,21,22,23,24]. STS process-oriented measurements often uses video recordings, to check the postures [23] or motion sequences [9,10]. Such protocols do not always control procedures as verbal instructions or the number of trials. All of these differences lead to non-standardized performance reporting. As far as we know, there are no studies that have established a full protocol for assessment of the STS task for all ages.

According to PICO strategy, the research problem presented in this study asked if, in typical development individuals (P), the assessment of the STS task performance (I) used in different developmental phases (C) can monitor healthy functional MC changes (O). In this context, the main objective was systematically reviewing the supine-to-stand task assessment methods and the health variables related to this task in youth, adults, and older adults. Specifically, it was examined: [1] The methods used to investigate the STS task, including the risk of bias, and the results, [2] the evidence of an association between the performance of the STS task and the health-related variables body weight status, musculoskeletal fitness, and physical activity. Finally, as a by-product of this review, a protocol for STS task measurement, which is testable across the lifespan, was proposed.

## 2. Materials and Methods

The present review included studies of the several designs (observational, interventional, cross-sectional, longitudinal), so it can be better classified as a mixed-methods review [25]. This study is registered in PROSPERO (#CRD42017055693) and followed protocols for systematic reviews PRISMA-P [26].

### 2.1. Data Sources and Searches Descriptors

**Figure 1:**
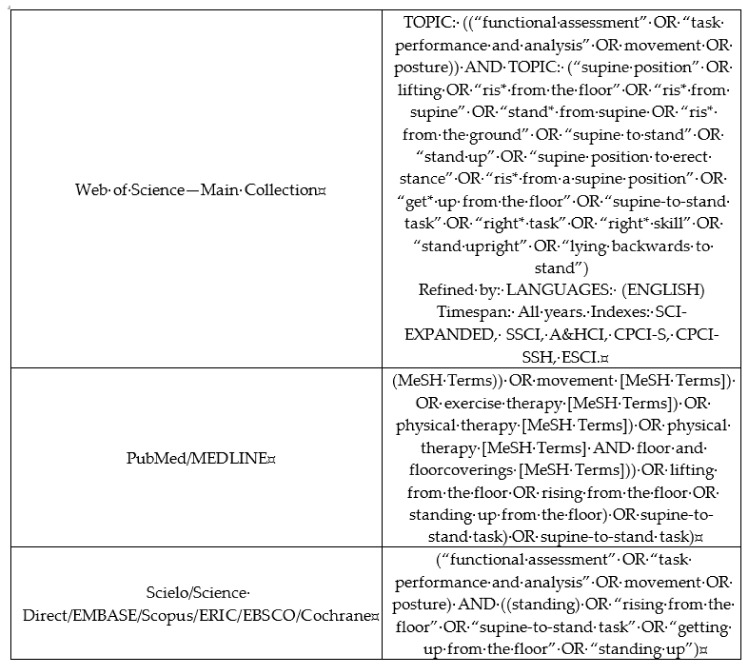
Descriptors used in the systematic review about the supine-to-stand (STS) task performance, according to research tools in the databases. The figure was originally created by the authors.

In this review, studies with healthy individuals in the phases of childhood, adolescence, adulthood, and senescence whose performance on the STS task had been assessed through objective measures, but not questionnaires, were searched. The databases were directedly accessed (MEDLINE, Scielo, EMBASE, Scopus, ERIC/ProQuest), and also it was used some search device (PubMed, Web of Science—Main Collection, Science Direct, EBSCO, Cochrane). Subsequently, the search involved gray literature, through the review of the reference lists (only of the included articles, see below) and consultation with specialists in the area. Intragroup descriptors were combined using the Boolean expression OR, whereas between-group descriptors were combined using AND (Figure 1). Inclusion criteria were: (1) Original studies (articles, theses, dissertations) assessing the STS task by using objective measures, (2) English language, (3) healthy/typical development individuals. Exclusion criteria were: (1) Duplicates, (2) not match with the background of this review, (3) not typical individuals, (4) articles not available in full text. There were no restrictions on the year of publication.

### 2.2. Study Selection

One author (MTC) conducted the identification of the studies, and added it to Rayyan QCRI, a web application for systematic reviews [27]. In this environment, the duplicates were removed by MTC. Before initiating the screening process, MTC and FSS performed an exhaustive training to include articles until they reached a concordance of the 92%; then, these two authors reviewed the list of titles for applying the inclusion criteria. So, the authors compared the results and discussed the discrepancies until they reach a consensus. If there was no consensus about a title, a third researcher resolved the disagreement (MPS). Such a process was repeated, reviewing abstracts and full texts, again applying the inclusion criteria. The identification of articles in the list of references and consultation with specialists supplemented the search strategy, added to the Rayyan application.

### 2.3. Data Extraction and Methodological Quality Assessment (Risk of Bias)

There was the extraction of the following data: Identification (author, year, and country of publication); objective, design, sample characteristics; STS outcome (process or product-oriented); results/conclusion strictly related to the STS task (Table 1). The number of trials, instruction for performance pace, participant’s caring, and motivational strategies from the protocols’ studies were also extracted (Table 2).

The quality of each article (risk of bias) was examined by 15 questions adapted from Law [28], and the scoring was as follows: 0 = does not meet criteria; 1 = satisfies the criteria; ? = not clearly described; NA = not applicable. In the classification, a study with score ≤ 7 = high risk of bias/low quality; studies that reached 8 to 11 points = moderate risk of bias/medium quality; scores ≥ 12 = low risk of bias/high quality (Supplementary material Table S1). Two researchers conducted these data extraction (MTC and FSS), and when there was no consensus, another researcher (ABD) resolved the disagreement.

The data were summarized in tables, and a narrative synthesis was done. Lastly, a full protocol was developed from the critical knowledge generated here. Some authors in this review worked in the clinical setting, and others are specialists in movement analysis, both in children and in the elderly. Then, the theoretical background and practical experience contributed to the presentation of this final protocol (Appendix A, Box A1).

## 3. Results

**Figure 2:**
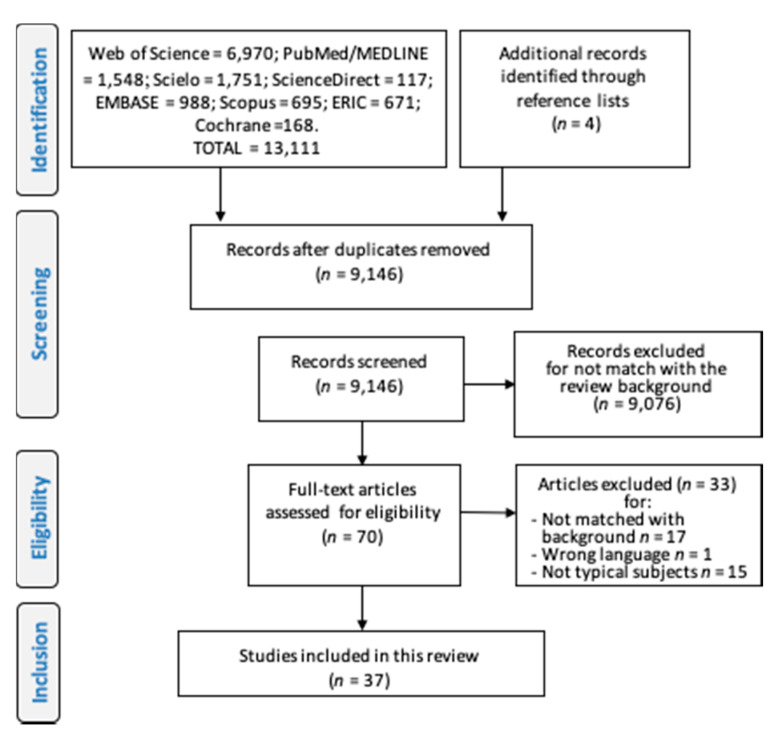
Flowchart describing the process to include studies in the systematic review according to the PRISMA-P protocol. The figure was originally created by the authors.

In this review, from of 13,155 studies found, 37 articles were included (Figure 2) [8,11,12,14,15,16,17,20,21,22,23,24,29,30,31,32,33,34,35]. The studies investigated subjects ranging in age from 1 to 94 years; the largest proportion of studies investigated up to 100 subjects [11,14,17,31,36,37,38,39,40], and one of them [41] examined a very large sample with 2368 subjects; more than a third of them used both process and product-oriented measurements (Table 1); only one study combined these two approaches creating a full score (MOD score) [40].

Just four studies reported the verbal instructions on starting position, which included legs extended and arms extended to the side of the trunk [14,31,36,40] five studies reported the final position as stable standing upright with both feet on the ground [23,24,31,38,40]. Nesbitt et al. [8] used final position goal combined with touching a designated point on the wall; Alexander et al. [4] asked the subjects to press a switch placed on a tripod when assuming the standing position, marking the end of the task. Other protocols’ characteristics were summarized in Table 2.

None of the studies that investigated the STS task were classified as high risk of bias. However, the item most frequently absent in assessing the risk of bias was “Was the justification for the sample size?”, with only 18.9% of studies presenting such a justification (for detailed information, see Supplementary Material Table S1).

### The STS Task Performance and Body Weight Status, Musculoskeletal Fitness and Physical Activity

By investigating young children, Ng et al. [31] and Nesbit et al. [12] did not find an association between STS time and BMI; by investigating older children, Duncan et al. [11] found a moderate inverse correlation (r = −0.508). Naugle et al. [37] investigated older adults and found that each unit of BMI was associated with an increase in the severity of compensatory strategies to rise; also investigating older people, Manckoundia et al. [38] found that being overweight was associated with fails to perform STS (not able to rise); Henwood and Taaffe [47] investigated the effect of a fitness program in seniors and found a positive effect on STS time. Belt et al. [42] investigated people from ages 7 to 36 years with Prader-Willi syndrome and typical controls, and regardless of diagnosis, they found a very poor correlation between STS task process measurement and BMI, but there were not confirmed relationships between BMI and any of the three body region scores. However, some cautions are need because BMI at different ages is related to various components of body composition.

Four studies investigated the relationship between STS task performance and musculoskeletal fitness, which showed a direct relationship [23,32,33,39]. One investigated children by testing grip and trunk muscle strength [32]; lower limb power of the older people were investigated [23,33,37,39], as well as flexibility [23] and upper limb strength [39]. One study [20] investigated a musculoskeletal training intervention on STS time performance in seniors that showed a positive effect.

Two studies examined the association between STS task performance with physical activity [14,39]. Green and Williams [14] investigated 72 middle-age adults and noted that the most active adults used more advanced STS task patterns than those who were rarely active, but they did not perform an inferential test. Klima et al. [39] investigated older adults and found an inverse correlation between STS task time and physical activity level (rho = −0.29).

## 4. Discussion

In this present review, the methods used in the STS assessment were summarized and critically examined. Furthermore, this review verified the association of STS task performance with select health variables. The results showed that the STS task performance was investigated throughout the life cycle, in various countries, and several studies used large samples [3,12,15,16,17,31,36,37,38]. In general, these results suggest the STS task can be considered a functional health assessment, from youth to old age.

However, the measurement type can be a critical issue. For example, by using a dichotomous variable (to be able or not gets up from the floor), Bergland et al. [15,16,17] investigated seniors (over 75 years) and concluded that the STS task is a valid marker of health and function problems, as well as a significant predictor of falls-related severe injuries in this life phase. One’s ability to stand up is a validated measure; however [15], it does not reveal the phenomenon of functional MC throughout life, since it seems to have been very suitable for use in the sample of older subjects, but it must have a ceiling effect at younger ones.

Both process and product-oriented measurements were used in a similar proportion in the literature. Process-oriented measurement identifies the difficulties in the task but demands much time to code and seems to be more valuable to propose intervention, mainly in older adults. Alternatively, product-oriented measurement, as movement time, maybe more related to functional outcomes. Facing a challenge, such as standing up as fast as possible, and relating this outcome to functional capabilities, like muscular strength and endurance, speaks to the ability to solve a functional motor task in various ways and at various speeds. This task speaks to the importance of being able to vary the execution of STS based on specific task demands. Thus, STS time is a resourceful way to operationalize functional MC, mainly with large samples or studies with many variables, as it requires limited technological resources and provides better discrimination and sensitivity in measurement than process-oriented assessments.

### 4.1. Risk of Bias and Procedure Protocols

Since all studies showed a moderate or low risk of bias, the internal validity was considered satisfactory. The more comprehensive analysis of the STS protocols showed a wide variety of procedures, which can be a severe problem if one proposes to have one protocol to be used for all the developmental levels. The instruction on the mechanics of the movement can facilitate performance [34,35] so for an understanding of how people typically get off the ground, controlling the instructions is critical to STS assessment. All studies instructed subjects individually, and most of them relied on verbal information rather than demonstration; some researchers even explicitly prevented any visual demonstration or explanation of the STS mechanics [11]. In summary, it was clear that researchers avoided any modeling or verbal instruction bias to examine the movement patterns typically used by participants.

Also, the time constraint instruction needs to be highlighted, since maximum speed instruction can affect the automaticity of the movements (i.e., minimizing the conscious analysis of the motor action) [53]. An external focus of attention (i.e., time restriction) organizes the motor system according to individual constraints and choice, rather than when the focus of attention is internal (i.e., on a movement pattern), which can interfere with the automatic process control as explained by the “constrained-action hypothesis” [35,53]. Depending on the measurement intents, a researcher can choose whether to impose a time constraint. For instance, to examine a general STS movement process that individuals would use in everyday life, a time constraint would not be necessary. Alternatively, to examine functional capacity as it relates to a “best” or “maximal” performance, a time constraint may be the most appropriate option. The time factor may be a more salient choice to predict health outcomes as the ability to produce power has strong implications for all-cause mortality and functional independence in older adults [54], as well as fitness, physical activity, and weight status in youth and young adults [5,55,56].

The final position of the task is another critical element related to the instructions. Two studies combined the goal of postural alignment with an external target [4,12]. This seems to be an efficient methodological strategy, since the performer has a simple and easy-to-execute external goal (touching the point in front of him/her), and in turn, the evaluators’ job is facilitated to stop the chronometer or video frame. However, understanding whether providing a final position with an additional reaching task might alter how an individual stand needs to be addressed.

Regarding feedback and rewards, three studies detailed the procedures given to motivate children by using verbal reinforcement during or after the task execution, using praise or words highlighting their efforts [3,12,24]. Motivational feedback was used only with early childhood children, since the motivational climate can dramatically affect preschoolers’ performance [55]. Encouraging may be highly beneficial if the time task constraint is “maximum” (i.e., shortest time) [40].

The number of repetitions varied widely among the studies. This lack of uniformity weakens the findings as a high number of repetitions without adequate rest can cause fatigue, adversely affecting motor performance. It seems to be the case with older or frail individuals that demonstrate limited physical function and fitness. Conversely, only one trial may not represent typical behavior. When addressing the movement process, two to five trials would be necessary to gain insight into an individual’s most probable movement process.

### 4.2. STS Performance and Body Weight Status, Musculoskeletal Fitness, and Physical Activity

Seven studies examined the body weight status and the STS task performance, and three of them demonstrating significant associations. It seems reasonable to expect that weight status, specifically with increased adipose tissue, is associated with STS performance. In overweight or obese individuals, the motor system has to overcome higher inertia to accelerate the body mass against the force of gravity to attain an upright position. Individuals can accomplish these using variable body actions that may not require high power outputs. Rather, maximizing postural alignments that minimize the demand for high muscle activity levels (i.e., manipulation of multiple degrees of freedom with minimum energy expenditure) would be a favorable strategy for individuals who demonstrate low muscular power/strength and endurance. However, while these strategies may be useful for minimizing energy expenditure, they may increase the time to stand. This potential trade-off may also speak to the variability in individuals’ MC. If individuals demonstrate higher MC levels, they may be able to stand using different coordination patterns regardless of energy used (i.e., level of neuromuscular demand or segmental coordination patterns), as demonstrated in Didier’s study [43]. However, individuals with low levels of overall MC may be more restricted in their movement patterns, due to a lack of muscular strength or coordinative capabilities across multiple joints. It is possible to think that the bodyweight status is a good candidate to be a moderator variable to the STS task performance, playing a role integrated with other fitness variables. More scientific pieces of evidence are necessary on this topic.

This review has confirmed there is a positive association between the STS task performance and musculoskeletal fitness for all ages, confirming previous literature results [5,6,55]. Therefore, it seems the STS is a good candidate to be a musculoskeletal health indicator in all cycle life.

The association between STS task performance and physical activity was examined in only two studies [14,39]. Even though the results had agreed with each other, and both studies have shown a low risk of bias, they have used different approaches, and one of them [14] did not report inferential associations. Thus, it seems too early to state that there is evidence of a direct association between physical activity and STS task performance. A previous study [56] has supported the notion of reciprocal action between the physical activity and MC, and perhaps it was the case of thinking more about how they enhance each other than just the simple relationship between them.

### 4.3. Clinical Applications

Hofmeyer et al. [20] carried out the only study that tested training for getting off the ground: Their results showed an improvement in the experimental group performance compared to the controls. This study reinforces that the STS task also has interventional value to health professionals. By taking all results of this review, one can generally state that STS is a potentially useful tool to examine functional MC and a general health status marker, as well as a useful approach to clinicians and researchers. It is notable in the present review that the STS task was investigated in the stages of childhood, adulthood, and old age. These results allow us to recognize that this is a task that is appropriate to play at all ages; in particular, a measure of the STS time has shown to have sufficient variability to distinguish individuals in all ages, without having a ceiling or floor effects.

The upright posture enables the subject to dominate his environment, and righting behaviors is an expression of physical independence [1]. Also, achieving such an upright posture in the shortest possible time, challenging individual constraints (e.g., unfavorable weight status), is a clear expression of human motor competence, i.e., the ability to solve motor problems in the face of challenging demands from the environment or of the subject itself. However, more evidence across the lifespan is needed to demonstrate these linkages.

Even the results of this review allow affirming that the STS task has a strong potential to uncover MC at any phase of life, the studies used diversified protocols and methodological strategies along with what prevented comparison and the generalization of findings. So, as a by-product of this review, a unique and universal protocol is proposed (Appendix A). We also understand that we are in agreement with contemporary literature on this subject. A very recent article [52] researched the same task with minor differences, showing its importance for older adults and investigating its clinimetric properties. We reinforce and extend the results of this study because we proposed that this task can be tested for all ages.

### 4.4. Study Strengths and Limitations

The strength of this study was that it examined all STS literature across the lifespan, and disentangled the various methods used to assess this task in several countries. It is plausible to present STS as a good candidate for a valid and non-culturally biased measure of functional MC across the lifespan. It is also advantageous to have a unique protocol that can be applied across all ages to facilitate tracking motor competence over time. The limitations of this study were that only studies in English were reviewed. Moreover, since the objective of this study was only to review methods, other studies should be carried out to establish typical values and significant cutoff points for the STS task performance.

## 5. Conclusions

This review showed that the STS task was tested at all ages, in various parts of the world, confirming it as a useful tool to track functional motor competence throughout life, in a universal way. In particular, measuring the time of the STS task is an ingenious way to operationalize functional motor competence mainly in large-scale studies. In addition, as it was found that the STS task has a strong relationship with musculoskeletal fitness over the years, it can help to monitor this health variable throughout life. It is not yet possible to recognize a factual relationship between the performance of the STS task and health variables, such as body weight status and physical activity.

## Figures and Tables

**Table 1:** Methodological characteristics, main results, and conclusions from studies on STS task (ordered by developmental phase and year of publication). The table was originally created by the authors.

Author (Year); Country	Objective	Study Design/Sample Characteristics	Main Outcome Measures		Main Results and Conclusions Related to STS Task
			Process or Product-Oriented Movement	Lifestyle/Motor Functions	
Childhood					
VanSant (1988) [3]; US	(1) To determine whether within the STS task MPs of different body regions vary with age, (2) describe movements used by children to perform this task.	OBS/pre-longitudinal/NP; 120 children divided into 4, 5, 6, and 7-year-old groups with 30 subjects each, matched by gender; G1 = 4.5 ± 0.27 yrs., G2 = 5.41 ± 0.28 yrs., G3 = 6.5 ± 0.27 yrs., G4 = 7.33 ± 0.24 yrs.	Process—MPs categories (UE, AX, and LE regions).	-	(1) Age differences in the incidence of MPs in each body region. (2) A trend toward increased symmetry of MPs as age increased. (3) The oldest subjects did not commonly use the symmetric form when rising. (4) Changes in the STS task likely to continue beyond early childhood.
Marsala and VanSant (1998) [24]; US	(1) To describe the MPs of toddlers on the STS task; (2) To determine whether toddlers’ MPs differ with age; (3) investigate whether MPs occur earliest in the development of this task prevail in toddlers.	OBS/pre-longitudinal/NP; 60 toddlers: 20 aged 15–25 months (mean age = 20.5 ± 2.9 mo.); 19 aged 26–36 months (mean age = 30.2 ± 2.6 mo.); 20 aged 37–47 months (mean age = 43.5 ± 2.6 mo.).	Process—MPs categories (UE, AX, and LE regions).	-	(1) Toddlers’ UE and AX movements confirmed previously developed MPs categories. (2) Age differences among toddlers regarding MPs. (3) MPs of UE and AX thought to occur earliest in the STS developmental sequence prevailed in this young group.
Mewasingh et al. (2002) [29]; Belgium	To analyze whether children with spastic diplegia use MPs as described for TD children and whether other MPs coexisted.	OBS/NP; 10 children with spastic diplegia associated with leukomalacia (♀ = 7; mean age = 7.5 ± 2 yrs.), CG: 14 age-matched TD children.	Process—MPs categories (UE, AX, and LE regions—adapted from Marsala and VanSant [12].	-	Children with spastic diplegia use MPs described in healthy children, but with markedly reduced within- and inter-individual variability.
Mewasingh et al. (2004) [30]; Belgium	To describe MPs in the STS task used by children with hemiplegic cerebral palsy (HCP).	OBS/NP; 15 children (♀ = 8; mean age = 7.3 ± 2.8) with HCP who were able to walk unsupported 5 m or more and perform the STS task without assistance; CG = 14 age-matched TD children.	Process—MPs categories (UE, AX, and LE regions, adapted from Marsala and VanSant [12]	-	Children with HCP performed the STS task using general MPs, but with reduced inter-individual variability compared to CG, with more asymmetrical patterns with systematic support on the unaffected side.
Beenakker et al. (2005) [36]; Netherlands	To report typical values for timed functional tests in TD children; to determine which parameter changes most in ambulant children with DMD by comparing typical values for muscle force and functional ability with values obtained by these children.	OBS/NP; 16 ambulant children with DMD (mean age = 6.25 ± 0.93); TD children: ♂ = 66 (mean age = 7.4 ± 2.3) and ♀ = 57 (mean age = 7.4 ± 2.2 yrs.).	Product—STS time (s).	Running 9 m.	STS time in TD children: 4 yrs. (♀ = 1.52 s; ♂ = 1.56 s), 5 yrs. (♀ = 1.45 s; ♂ = 1.45 s), 6 yrs. (♀ = 1.17 s; ♂ = 1.42 s), 7 yrs. (♀ = 1.19 s; ♂ = 1.28 s), 8 yrs. (♀ = 1.03 s; ♂ = 1.24 s), 9 yrs. (♀ = 1.06 s; ♂ = 1.17 s), 10 yrs. (♀ = 1.42 s; ♂ = 1.08 s), and 11 yrs. (♀ = 1.13 s; ♂ = 0.99 s). The DMD children’s performance declined with age, while TD children improved it; DMD children (8 yrs.) took 7.5 times longer than DD ones; timed functional testing seemed to be more sensitive to determine disease progression and functional impairment changes than force measurement.
Ng et al. (2013) [31]; UK	To define typical values from the time to STS and the time to run 10 m, formulate charts for these tests, and assess their reproducibility.	OBS/pre-longitudinal screening/NP; n = 321 TD children; ♀ = 160; age range = 2–8 yrs. (mean age = 5.1 yrs.).	Product—STS Time (s); Process—the method used to stand from the supine position.	Age, sex, height, weight, BMI, and time to run 10 m.	STS time = 2.08 s (range 1.03–5.28 s); an association between the standing method and age; boys: Association between the standing method and STS time; large variability in the method used and STS time in youngers; strong negative correlation with age; height, weight, or BMI not affected the STS time; charts showed age-related values.
Kuwabara et al. (2013) [32]; Japan	To determine the relationship between the choices of movement patterns in STS task and physical functions in healthy children. O	68 TD children (♀ = 42), age range = 3.4–6.4 yrs.	Process—MPs categories (UE, AX, and LE regions, from VanSant [3]	Age, sex, grip strength, trunk flexor, and extensor strength; balance in one-leg standing time(s)	Children who demonstrated symmetrical MPs had significantly higher grip and trunk muscle strength and better balance than children who showed asymmetrical MPs. The symmetrical MPs were explained by a positive relationship to grip strength and trunk flexor strength. Muscle strength seems to be related to symmetrical MPs of the STS task in healthy children.
Hsue (2014) [21]; Taiwan	To determine the within—and inter-rater reliability in classifying the MPs of STS task in TD children and children with mild to moderate DD.	OBS/NP; 68 TD children: 5–6 yrs. (n = 15), 4–5 yrs. (n = 19), 3–4 yrs. (n = 20), and 2–3 yrs. (n = 14); 20 children with DD: 5–6 yrs. (n = 4), 4–5 yrs. (n = 4), 3–4 yrs. (n = 6), and 2–3 yrs. (n = 5).	Process—MPs categories (UE, AX, and LE regions)	Developmental capability tested by Peabody Developmental Motor Scale-II.	Complexities and difficulties affecting the within- and inter-rater reliability in classifying the MPs of STS task were related to developmental capability, age, and body region. Extra training seems to be needed for children with DD, particularly for the UE and LE regions.
Hsue (2014) [22]; Taiwan	(1) To determine MPs of children with DD used to STS task and how they differ from age-matched TD children, (2) to verify whether MPs differ with age in children with DD, and (3) To determine and compare the developmental sequences for the MPs for UE, AX, and LE in DD children and TD children.	OBS/NP; 66 TD children: 5–6 yrs. (n = 15), 4–5 yrs. (n = 19), 3–4 yrs. (n = 19), 2–3 yrs. (n = 13); 31 children with DD: 5–6 yrs. (n = 5), 4–5 yrs. (n = 6), 3–4 yrs. (n = 8), 2–3 yrs. (n = 12).	Process—MPs categories (UE, AX, and LE regions) from VanSant [3] revised by Marsala and VanSant [12], and Belt et al. [40]	Developmental capability tested by Peabody Developmental Motor Scale-II (	The TD group followed the proposed developmental sequences, as well as the DD group, which showed different maturation speed and more variability, especially between the age of 3 and 5 yrs.; the most used MPs by children with DD were at least one developmental categorical pattern behind those used by the age-matched TD children before 5 yrs. Old, except for the LE region. In the DD group, children with better motor performance used more developmentally advanced patterns.
Duncan et al., (2017) [11]; UK	To examine how STS performance is related to process and product assessment of motor competence (MC) in children.	OBS/NP; 91 TD children (♀ = 44) aged 5–9 years (mean age = 6.8 ± 1.2 yrs.).	Product—STS Time (s) Process—MPs categories (UE, AX, and LE regions—VanSant [3].	MC score comprised four skills: run, jump, catch, throw, 10 m running speed, and standing long jump distance.	Children who scored higher on STS process also scored higher on MC process and were faster in the 10 m running time; a significant association between STS time and BMI (r = −0.508), STS time and STS process (r = −0.463), standing long jump distance (cm) and STS time (r = −0.414), and 10 m running speed (s) and STS time (r = 0.539). STS test is a measure of functional MC in children.
Nesbitt et al. (2017) [12]; US	To examine the relationship between qualitative (developmental sequences) and quantitative (time) performance rising from a supine position in early childhood.	OBS/NP; 122 TD children (♀ = 56); age range = 3–5 years (mean age = 4.63 ± 0.5 yrs.).	Product—STS Time (s) Process—MPs categories (UE, AX, and LE regions—VanSant [3].	BMI	The children’ STS task performance was quite variable in terms of qualitative MPs; STS mean time = 2.37 s, ± 0.60. The levels of the components of UE (r = -.383) and AX (r = −416) were correlated with time. Results indicated a strong association between trunk control development and UE (r = 0.791) movement levels, and together they demonstrated the strongest effect on STS performance. There was no association between BMI and time in the STS task.
Childhood and Adulthood					
Belt et al. (2001) [42]; US	To determine if previously published descriptors of the STS task in healthy individuals could be applied to the movements of persons with Prader-Willi Syndrome; and 2) assess UE, AX, LE region movements among subjects with PWS compared with TD controls.	OBS/NP; 9 subjects (children and adults) with PWS and nine matched TD controls; age range = seven –36 yrs.	Process—MPs categories (UE, AX, and LE regions) were classified using modified descriptors developed by Marsala and VanSant [12]; Product—STS time (s)	BMI	Subjects with PWS utilized less advanced asymmetrical rising patterns, took longer to rise (5.4 s for subjects with PWS and 2.86 s for controls), and demonstrated less within-subject variability than controls. Knowledge of successful rising patterns may use to assess and plan intervention strategies. Regardless of diagnosis, there was a weak correlation between body region movement score and BMI (rho = 0.01). There was no relationship between BMI and body region scores.
Nesbitt et al. (2018); [8]; US	To examine the validity of STS as a developmental measure of functional MC across childhood into young adulthood and examining associations between movement components. STS time also provided a secondary measure of developmental validity in addition to an examination of the concurrent validity of STS against developmentally valid measures of MC.	OBS/NP; 265 subjects ♂♀ (children and adults) distributed in 4 age groups: 3–6 (mean age 4.8 ± 0.9 years); 9–12 (mean age 10 ± 0.8 years); 13–17 (mean age 14.9 ± 0.9 years); 18–25 (mean age 20.9 ± 2 years)	Process—MPs categories (UE, AX, and LE regions) were classified using modified descriptors developed by Marsala and VanSant [12]; Product –STS time (s)	Developmentally valid measures of motor competence Throwing, kicking, jumping, hopping, and STS skills test	Results indicated that cross-sectional curves for the STS components generally fit Roberton’s (1980) hypothetical model curves. STS time demonstrated weak to moderate correlations to STS time across all age groups, indicating that it can be considered a valid and reliable measure of MC across childhood into young adulthood
Adulthood					
VanSant (1988b) [10]; US	(1) To describe MPs within specific body regions used to stand up from a supine position. (2) To identify motor developmental sequences for the UE, AX, and LE regions during this rising task.	OBS/NP; 32 healthy adults (♀ = 17); age range = 20–35 yrs. (mean age = 28.6 yrs.).	Process—MPs categories (UE, AX, and LE regions).	-	Subjects varied greatly in the MPs; 25% of subjects demonstrated a similar combination of MPs during rising, which involved the symmetrical use of the limbs and trunk while flexing forward from a supine position, moving through sitting to squatting, and then standing. An ordering of categories for each body region was proposed as a developmental sequence of STS MPs.
Green and Williams (1992) [14]; US	(1) To validate categories for the MPs of STS task in adults. (2) To evaluate the influence that physical activity might have on the MPs used for rising.	OBS/NP; 72 adults, age range = 30–39 years (mean age = 34.1 ± 2.8 yrs.) divided into three groups: group 1 (n = 25) reported daily physical activity, group 2 (n = 26) reported exercising once or twice a week, and group 3 (n = 21) reported did exercises less than once a week.	Process—MPs categories (UE, AX, and LE regions—VanSant, [3].	Level of physical activity (questionnaire).	More active people used more advanced MP than the rarely active ones. The lifestyle patterns of regular, moderate physical activity may influence the STS task performance. This study provided support for the use of developmental sequences for the MP of the STS task.
Didier et al. (1993) [43]; France	To compare the energetic costs of daily activities in young and older adults, such as rising and sitting back down on a seat, getting up from and lying down on a bed, and getting up from the floor.	OBS/NP; 10 healthy men (mean age = 24.3 ± 2.8 yrs.), and 10 older men (mean age = 74.4 ± 2.2 yrs.).	Product—STS time (s).	Energy Cost	Getting up from and lying down on the floor or a standard hospital bed involved the same energy cost in the older and younger group, but performing these activities took significantly longer for the older people
King and VanSant (1995) [41]; US	To verify if SAFOs affect the MPs used in the STS task, and to determine the mode and the incidence of MPs under each condition.	Interventional/NP convenience sample; 39 healthy adults, age range = 20–28 yrs. (mean age = 22.7 ± 1.87 yrs.).	Process—MPs categories (UE, AX, and LE regions).	-	Changes in the incidence of MPs occurred in all SAFO conditions, but not with the no SAFO condition. Changes resulted in more asymmetry in SAFOs condition, mainly in the axial region.
Adulthood and Elderly phase					
Alexander et al. (1997) [4]; US	(1) To determine the ability of older adults to rise from the floor; (2) explore how the ability to rise might differ based on the initial body position, with and without the use of an assistive device.	OBS/NP; 24 (♀ = 12) adults, age range = 19–30 years (mean age = 23 yrs.); 24 (♀ = 12) healthy older adults, age range = 66–87 yrs. (mean age = 73 yrs.); 38 (♀ = 32) older adults, living in congregate housings for the elderly, age range = 63–94 yrs. (mean age = 80 yrs.).	Product—STS time (s); Process—from five different initial positions, with and without external support: (1) supine; (2) on the side; (3) prone; (4) all fours; and (5) sitting.	Perceived level of difficulty.	Older adults had more difficulty performing STS task than young. Healthy older adults took two times longer than adults to rise; congregate older adults took 2–3 times more than healthy older adults. Adults and healthy older adults rose from every position; Congregate older adults were most likely rising successfully from a side-lying position using furniture for support. The most capable subjects rose more quickly and had fewer difficulties when rising from the all-fours position.
Ulbrich et al. (2000) [44]; US	To describe how older adults, particularly more physically impaired older adults, might differ from healthy young adults in the body positions used to rise from the floor.	OBS/NP; 22 (♀ = 11) young adult controls, age range = 19–30 yrs. (mean age = 23 ± 3; yrs.), 24 (♀ = 12) healthy older adults, age range = 66–87 yrs. (mean age = 73 ± 6 yrs.), and 29 (♀ = 29) congregate housing older adults, age range = 63–94 yrs. (mean age = 81 ± 7 yrs.)	Product—STS time(s); Process—Intermediate Position (IP): sit, crouch, side-lying, tuck, half-tuck, kneel, half-kneel, crouch-kneel, all fours, bear walk.	-	Congregate residents were slowest in rising (17.1 s) and used the most IP, followed by healthy older adults (5.5 s) and young controls (2.6 s). The most preferred rise strategy used by controls was sat and crouch, whereas congregate residents used tuck, crouch-kneel, all fours, and bear walk; healthy older adults used IPs common to young adults and congregates
Bohannon and Lusardi (2004) [33]; US	(1) To explore the relationship between STS performance and age, functional lower extremity strength, and balance; and (2) to describe movement strategies used by healthy older adults when getting up from the floor.	OBS/NP; 52 (♀ = 38) healthy and independent community-living volunteers, age range = 50–90 yrs. (mean age = 64.6 ± 9.5 yrs.). There was a relatively equal distribution of participants across decades of ages within the sample.	Product—STS time (s); Process—three distinct stages: Initiation, transitional weight transfer, and going to upright posture, and there were some strategies in each stage.	Muscle strength: Time to complete five sit-to-stand cycles. Balance: Timed (s) single limb stance with eyes open (up to 30 s) both sides.	STS time = 4.1 ± 1.1 s, ranging from 1.8 to 7.2 s; Correlations: STS time and age, r = 0.48; STS time and sit and stand test, r = 0.64; STS time and single right stance, r = −0.36; STS and left single stance, r = −0.42; STS performance may be enhanced by training that addresses impairments in lower extremity strength and balance.
Schwickert et al. (2015) [23]; Germany	(1) To develop a model of MP sequences for unassisted STS task from different lying positions; (2) to identify differences in the MPs and transfer times of healthy older adults compared to healthy young adults, and identify difficulties in the MPs of older adults; and (3) to verify the associations with executive function, power, and flexibility.	OBS/NP; 14 (♀ = 7) young adults, age range = 19–39 yrs., and 10 (♀ = 5) older adults, age range = 59–79 yrs.	Product—STS time (s); Process—type and number of components to perform the STS task in a naturalistic scenario and a standardized scenario.	Trail making test; maximum gait speed 4 m distance, 30 s chair rise, Romberg test, Nottingham power rig, chair sit-and-reach test, goniometry.	Seven task components were noted: Lying, initiation, positioning, supporting, and elevation stabilization followed by quiet stance or walking; older adults = 5.7 s vs. young adults = 3.7 s (*p* < 0.001). There was a reduction in STS performance in older subjects, which was associated with reduced power and flexibility. Executive function, leg and hip power, and knee flexibility were lower in the older adult group. The scenario type did not influence the number of STS task components.
Schwickert et al. (2016) [45]; Germany	To analyze different kinematic features of standing up from the floor in adults and healthy older adults using inertial sensors describing such transfer patterns.	OBS/NP; 14 (♂ = 7) adults, age range = 20–50 yrs., and 10 (♂ = 5) healthy older community dwellers aged ≥60 yrs.	Product—Transfer time (s); transfer angular velocity; vertical velocity and acceleration; jerk Process—smoothness, fluency, and complexity of movement strategies.	-	The motion sequences of the older adults were less fluent and smooth than in the younger group; older subjects used more indirect movement strategies, including more turns around the longitudinal axis to prepare for elevation. There was the feasibility of describing and discriminating the performance kinematics of younger and older subjects standing up from the floor from different lying postures, using inertial sensor signals at the trunk.
Elderly					
Schenkman et al. [46] (2000); US	To determine (a) the associations between spinal flexibility and functional limitations; (b) the relative contribution of spinal flexibility to specific functional limitations; and (c) how disease state (PD vs. no PD) modified these relationships	OBS/NP; n total = 251; 56 older adults with PD (♀ = 24,5%) (mean age = 70.7 ± 7.4 yrs.); 195 non-PD (mean age = 71.4 ± 5.0 yrs.)	Product—STS time (s);	Spinal flexibility; functional reach distance; 10-m walk time; number of steps to turn 360°	PD older adults, STS time = 7.2 ± 3.7; non-PD older adults, STS time = 5.2 ± 2.0. Spinal flexibility was a significant predictor of supine-to-stand time and the number of steps in the 360 degrees turn, but there was no clinical significance for these two variables
Bergland et al. (2002) [15]; Norway	To evaluate the concurrent and prospective validity of self-reported items concerning walking and balance.	OBS/Probabilistic, longitudinal, and predictive; 307 elderly women, living at home, age range = 75–93 yrs. (mean age = 80.8 yrs.).	Product—scored according to whether the subject managed to perform the STS task without assistance (1 point) or not (0 points).	Tandem stance/eyes open; functional reach; one-legged stance/eyes open; walking in figure-eight; climbing stairs; self-reported walking difficulties.	About 80% of the women managed to perform the STS task and could cope with steps higher than 30 cm; younger subjects performed better than those in the higher age bands in all tests; all clinical tests correlated significantly with each other (range = 0.25–0.85) and also with the self-reported walking index (range = 0.32–0.62).
Hofmeyer et al. (2002) [20]; US	To determine the effect of a 2-week training intervention to improve disabled older adults’ ability to rise from the floor.	Interventional/Random allocation; healthy older adults. Training group (n = 17, ♀ = 13; mean age = 81 ± 6 yrs.) submitted to an individual training in strategies to rise from the floor using key body positions; control group (n = 18, ♀ = 13; mean age = 80 ± 7 yrs.) submitted to an chair-based flexibility intervention.	Product 1—able or not able to perform the STS task in eight different conditions; Product 2—STS time.	The Perceived Scale of Symptoms and Difficulties.	The training group showed a significant improvement in the post-test mean number of rising tasks completed; regarding the supine position, the mean rise time varied from 21–25 s at baseline to 20–27 s post-intervention, but such improvement was not significant. The training group showed a significant improvement in the level of difficulties and symptoms.
Bergland et al. (2004) [16]; Norway	To verify whether balance, function, and other health status indicators can predict serious fall-related injuries or fall-induced fractures in older women.	OBS/Probabilistic, longitudinal, and predictive; 307 women, age range = 75–93 yrs. (mean age = 80.8 yrs.) who were living at home.	Product—scored whether the subject managed to perform the tasks without assistance (1 point) or not (0 points).	Other measures: Serious fall injuries over a year, health records, function, and walking and balance	Rheumatic disorders and the inability to perform STS task were the most substantial independent risk factors for fall-related severe injuries.
Henwood et al. (2005) [47]; Australia	To investigate the effects of a short-term high-velocity varied resistance training program on physical performance in healthy community-dwelling older adults aged 60 to 80 years.	Interventional/NP; 25 healthy community-dwelling men and women, age range = 60–80 yrs.; they were divided in two groups: Experimental (n = 14; 69.9 ± 6.5 yrs.) and control group (n = 10; 71.3 ± 5.6 yrs.).	Product—STS time (s).	Muscle power and strength measures: Chair rise to standing, 6-m walk, lift and reach; BMI and percentage of body fat.	In Baseline, Experimental group STS time = 4.5 ± 0.8 s; control group STS time = 3.8 ± 0.9 s; after training there was 10.4% reduction in time in the experimental group from the baseline (*p* = 0.006). There was a group X time interaction for floor rise to standing (experimental group). There was no change in body composition during the study.
Bergland et al. (2005) [17]; Norway	To assess the concurrent and predictive validity of older women’s ability to get up from lying on the floor.	OBS/Probabilistic; predictive; 307 elderly women, age range = 75–93 yrs. (mean age = 80.8 yrs.) who were living at home.	Product—scored whether the subject managed to perform the tasks without assistance (1 point) or not (0 points).	Falls and falls-related injuries, function measures, and health and social resources.	The STS task is a valid marker of failing health and function in older adults and a significant predictor of serious fall-related injuries.
Manini et al. (2006) [40]; US	Develop a task modification scale to examine its reliability and comparability to timed performance and standard measures of physical function and impairment in older adults	OBS/NP; 82 (♀ = 21) older adults (mean age = 74.4 ± 8.2 yrs.)	Product—STS timeProcess—MOD Score	Gait speed (fast and regular), five chair rises, self-reported physical function, knee extensor strength, and single-leg balance.	The MOD score is reliable across raters and repeatable within participants; also, it showed higher correlations with muscle strength and balance impairment than did other measures as gait speed, time to complete five chair stands, and self-reported physical function.
Mankoundia et al. (2007) [48]; France	To determine whether the management, including medical, psychological and physiotherapeutic approaches may be beneficial in the short and medium-term, for elderly fallers with psychomotor disadaptation syndrome.	Interventional/NP; longitudinal; 28 (♀ = 25) elderly fallers (mean age = 81.43 ± 6.7 yrs.).	Product—STS time (s)	Functional Independence Measure, Mini Mental State Examination, Tinetti test, Mini Motor test, Dual Task test, Beck Depression Inventory-II, Covi Scale, Modified-Falls Efficacy Scale	The multidisciplinary intervention had an overall positive impact on motor abilities as shown by the increase in the mini-motor test scores, the rate of success in rising from the floor and decrease of time for the dual task.
Manckoundia et al. (2008) [38]; France	Identifying the demographic and clinical parameters, assessed during a standard health examination, affects balance control in older adults.	OBS/NP; 2368 (♀ = 1215) older adults (mean age = 70.0 ± 4.5 yrs.).	Product—able or unable to perform the STS task.	Age, gender, BMI, cognitive status, self-perception of health, and use of psychotropic drugs	Women (8%) failed more than men (2.7%) in the STS task. Women who failed in the STS task had higher diastolic blood pressure and glycemia; BMI and health scores determined errors in the STS task in both genders. The BMI was a significant determinant of performance in all balance tests.
Geraldes et al. (2008) [49]; Brazil	To investigate the relationship between flexibility of flexion and extension of the glenohumeral and coxofemoral joints and functional performance among physically active and functionally independent elderly women.	OBS/NP/22 functionally independent elderly women mean (age 70 ± 6 yrs)	Product-STS time (s).	Flexibility of the glenohumeral and hip joints	There was a significant association between assisted-active flexibility and STS performance.
Naugle et al. (2012) [37]; US	To examine the association between compensatory strategies to successfully daily activities and body mass in pre-clinically disabled older adults.	OBS/NP; 259 (♀ = 116) older adults (mean age = 67.6 ± 7.0 yrs).	Process—MOD Score evaluated (0 to 5 points) participants’ performance on each task according to the severity of the compensatory strategy to complete the task.	Chair rise from three heights (43, 38, and 30 cm), kneel to stand, stair ascent, stair descent, and lift and carry a basket filled with 10% of the subject’s BMI.	The obese class II group had a higher likelihood of using one or more compensatory strategies while performing the STS task compared to all other groups. Individuals categorized as overweight and obese Class-I were more likely to use compensatory strategies while performing the STS task than the healthy weight group.
Raso and Greve (2012) [50]; Brazil	To determine the effect of an aerobic or resistance exercise protocol on the performance of daily living activities in older women.	Interventional/NP/random allocation; 41 healthy elderly women, age range = 60–85 yrs. (mean age = 65.1 ± 7.9 yrs.) randomly assigned to resistance (n = 22) or aerobic exercise (n = 19).	Product—STS velocity (s).	Performance in these tasks was measured while subjects were wearing sneakers: Sitting to standing position, STS task, and climbing stairs.	Subjects of the aerobic exercise protocol improved speed significantly when wearing sneakers, while subjects of the resistance exercise protocol improved their performance in the STS task and climbing stairs when using these shoes.
Klima et al. (2016) [39]; US	To examine physical performance correlates of timed supine to standing performance, Furthermore, to identify the predominant motor pattern used to complete rising from the floor.	OBS/NP; 53 (♀ = 36) older adults (mean age = 78.5 ± 8.5 yrs.).	Product—STS time (s); Process (symmetrical rise and squat sequence or symmetrical rise and asymmetrical squat sequence or roll and push maneuver).	Handgrip, balance, 9-m walk test, TUG test; Physical Activity Scale for Elderly	STS time was associated with age (r = 0.57), gait velocity (r = −0.61), ABC scores (r = −0.51); there were correlations with physical activity (Rho = −0.29), grip strength (r = −0.30); and with the TUG test (r = 0.71). Hierarchical regression demonstrated that TUG performance predicted 48% of the variance in STS time (*p* < 0.001). This significance remained after adjusting for age and BMI covariates.
Manckoundia et al. (2020) [51]; France	To investigate the impact of an ambulatory physical activity program on the motor skills of retirees	Interventional, not controlled study/NP. N total = 200 living home healthy older adults (♀ = 172), age range = 60–100 yrs. (mean age = 73.8 ± 7.4 yrs.). They were divided into two groups for STS task: Robust subjects vs. frail or very frail subjects. The program included strengthening muscular and joint flexibility exercises, balance work, one-leg-balance test, stimulation of the foot arch, STS task, TUG, gait speed, one-leg-balance	Product—able or unable to perform the STS task.	One-leg-balance test, TUG, gait speed, one-leg-balance test duration	For STS, 81% of participants did not change groups after training program, 18.5% changed from (very) frail to robust, and 0.5% of subjects changed from robust to (very) frail.
Moffett et al. (2020) [52]; US	To describe the performance and clinimetric properties STS completed by apparently healthy community-dwelling older women	OBS/methodological quality study; 52 ♀ (mean age = 66.4 ± 8.1 yrs)	Product—STS time (s)	36-Short Form Health Survey, Gait Speed test, Sit-To-Stand test, Johns Hopkins Fall Risk Assessment	STS test appears to be informative, valid, and reliable, at least for older independent women.

♀ = female; ♂ = male; AX—Axial region; BMI—Body Mass Index; CG—Group control; DD—Development delay; DMD—Duchenne muscular dystrophy; DS—Developmental sequence; HPC—Hemiplegic cerebral palsy; IP—Intermediate Positions; LE—Lower extremity; MCR—Medical Council Research scale; mo.—months; MOD Score—score representing task modifications; MPs—Movement Patterns; NP—non-probabilistic; OBS—observational study; PD—Parkinson’s disease; PWS—Prader-Willi Syndrome; SAFOs—Solid ankle-foot orthoses; STS task—Supine to Stand task; STS Time (s)—Time needed to complete the STS task in seconds; TD—Typical development; TUG—Timed Up and Go test; UP—Upper extremity; yrs.—years. Source: the authors.

**Table 2:** Absolute and relative frequencies of the protocols’ characteristics of studies reviewed about the Supine-to-Stand (STS) task (*n* = 37).

		N	*f (%)*
Quality of study	Superior–Low risk of bias (≥ 12)	18	64.9
	Medium–Moderate risk of bias (8 to 11)	13	35.1
	Inferior–High risk of bias (≤ 7)	0	0.00
Number of trials *	Only one trial	7	18.9
	2 to 5 trials	12	32.4
	6 to 10 trials	10	27.0
	Above 10 trials	2	5.4
Instruction for performance speed	“As fast as possible”	18	48.6
	Comfortable speed	14	37.8
	Not informed	4	10.8
Participant’s caring *	Use of the test trail	32	86.5
	Rest interval	11	29.7
	Demonstration	6	16.2
	Use of the assistants	6	16.2
Motivational strategies *	Feedbacks	3	8.1
	Rewards	1	2.7

* Not all studies have described these procedures. The table was originally created by the authors.

## Supplementary Materials

The following are available online at https://www.mdpi.com/1660-4601/17/16/5794/s1, Table S1 Risk of bias within articles included in the review regarding the performance of the supine-to-stand (STS) task.

## Appendix A

Box A1 Supine-To-Stand (STS) task measure protocol for all ages. Objective: To get up as fast as possible from the floor. Place and procedures 1. Quiet room with a clean and flat floor; 2. Floor marks. With a contrasting colored adhesive tape mark on the floor a perpendicular distance equal to the participant’s arm length to the wall; this distance cannot be less than 30 cm, approximately the same distance of the semi-extend arms to the wall, in order to be a comfortable space to the participant rising him/herself. 3. Mark a dot on the wall. The placement of the target on the wall must match with the height of the subject’s gaze. 4. Initial position: Lie flat (dorsal decubitus position) on the ground with hands in a prone position; 5. Final position: Upright orthostatic position touching a fixed target on the wall. General recommendations

✓ The participant’s feet must be bare, and it is suggested that participants wear light clothes, suitable for exercise;
✓ The subject makes a trail test in order to train before performing valid attempts.
✓ All commands given to participants must be communicated with kindness and firmness;
✓ Do not give any feedback to participants between tests;
✓ Do not give any visual instructions;
✓ Repeat a minimum of three times and more if needed to address process-oriented mode sequences; the best performance (lowest movement time) is the primary measure for the analyses;
✓ The interval between trials must last for as long as it takes for the participant to reposition himself/herself on the floor. It is not a problem if the participant takes more time between one attempt and another to return to the initial position: Individual comfort should be maintained;
✓ In the case of elderly participants, at least one assistant must be behind the performer to provide any help during the action of rising from the ground.

## References

[1] VanSant A.F. (1990). Life-span development in functional tasks. Phys. Ther..

[2] Leversen J.S.R., Haga M., Sigmundsson H. (2012). From children to adults: Motor performance across the life-span. PLoS ONE.

[3] VanSant A.F. (1988). Age Differences in Movement Patterns Used by Children to Rise from a Supine Position to Erect Stance. Phys. Ther..

[4] Alexander N.B., Ulbrich J., Raheja A., Channer D. (1997). Rising from the floor in older adults. J. Am. Geriatr. Soc..

[5] Cattuzzo M.T., dos Santos Henrique R., Ré A.H.N., de Oliveira I.S., Melo B.M., de Sousa Moura M., de Araújo R.C., Stodden D. (2016). Motor competence and health related physical fitness in youth: A systematic review. J. Sci. Med. Sport.

[6] Robinson L.E., Stodden D.F., Barnett L.M., Lopes V.P., Logan S.W., Rodrigues L.P., D’Hondt E. (2015). Motor Competence and its Effect on Positive Developmental Trajectories of Health. Sport. Med..

[7] Stodden D.F., Langendorfer S.J., Goodway J.D., Roberton M.A., Rudisill M.E., Garcia C., Garcia L.E. (2008). A developmental perspective on the role of motor skill competence in physical activity: An emergent relationship. Quest.

[8] Nesbitt D., Molina S.L., Sacko R., Robinson L.E., Brian A., Stodden D. (2018). Examining the Feasibility of Supine-to-Stand as a Measure of Functional Motor Competence. J. Mot. Learn. Dev..

[9] VanSant A.F. (1988). Rising from a supine position to erect stance. Description of adult movement and a developmental hypothesis. Phys. Ther..

[10] Duncan M.J., Lawson C., Walker L.J., Stodden D., Eyre E.L.J. (2017). The Utility of the Supine-to-Stand Test as a Measure of Functional Motor Competence in Children Aged 5-9 Years. Sports.

[11] Nesbitt D., Molina S.L., Cattuzzo M.T., Robinson L.E., Phillips D., Stodden D. (2017). Assessment of a Supine-to-Stand (STS) Task in Early Childhood: A Measure of Functional Motor Competence. J. Mot. Learn. Dev..

[12] Marsala G., VanSant A.F. (1998). Age-related differences in movement patterns used by toddlers to rise from a suppine position to erect stance. Phys. Ther..

[13] Green L.N., Williams K. (1992). Differences in developmental movement patterns used by active versus sedentary middle-aged adults coming from a supine position to erect stance. Phys. Ther..

[14] Bergland A., Jarnlo G.B., Laake K. (2002). Validity of an index of self-reported walking for balance and falls in elderly women. Adv. Physiother..

[15] Bergland A., Wyller T.B. (2004). Risk factors for serious fall related injury in elderly women living at home. Inj. Prev..

[16] Bergland A., Laake K. (2005). Concurrent and predictive validity of “getting up from lying on the floor”. Aging Clin. Exp. Res..

[17] Hands B.P., Hands B. How Can We Best Measure Fundamental Movement Skills?. Paper presented at the Australian Council for Health, Physical Education and Recreation Inc. (ACHPER) 23rd Biennial National/International Conference: Interactive Health & Physical Education.

[18] Burton A.W., Miller D.E. (1998). Movement Skill Assessment.

[19] Hofmeyer M.R., Alexander N.B., Nyquist L.V., Medell J.L., Koreishi A. (2002). Floor-rise strategy training in older adults. J. Am. Geriatr. Soc..

[20] Hsue B.J., Chen Y.J., Wang Y.E. (2014). The intra- and inter-rater reliability of component analysis of rise from supine in the children with typical development and developmental delay. Res. Dev. Disabil..

[21] Hsue B.J., Wang Y.E., Chen Y.J. (2014). The movement patterns used to rise from a supine position by children with developmental delay and age-related differences in these. Res. Dev. Disabil..

[22] Schwickert L., Oberle C., Becker C., Lindemann U., Klenk J., Schwenk M., Bourke A., Zijlstra W. (2016). Model development to study strategies of younger and older adults getting up from the floor. Aging Clin. Exp. Res..

[23] Grant M.J., Booth A. (2009). A typology of reviews: An analysis of 14 review types and associated methodologies. Health Info. Libr. J..

[24] Moher D., Liberati A., Tetzlaff J., Altman D.G., Altman D., Antes G., Atkins D., Barbour V., Barrowman N., Berlin J.A. (2009). Preferred reporting items for systematic reviews and meta-analyses: The PRISMA statement. PLoS Med..

[25] Ouzzani M., Hammady H., Fedorowicz Z., Elmagarmid A. (2016). Rayyan-a web and mobile app for systematic reviews. Syst. Rev..

[26] Law M., Stewart D., Letts L., Pollock N., Bosch J., Westmorland M. (1998). Guidelines for Critical Review of Qualitative Studies.

[27] Mewasingh L.D., Demil A., Christiaens F.J.C., Missa A.M., Cheron G., Dan B. (2002). Motor strategies in standing up in leukomalacic spastic diplegia. Brain Dev..

[28] Mewasingh L.D., Sékhara T., Pelc K., Missa A.M., Cheron G., Dan B. (2004). Motor strategies in standing up in children with hemiplegia. Pediatr. Neurol..

[29] Ng J., Conaway M.R., Rigby A.S., Priestman A., Baxter P.S. (2013). Methods of standing from supine and percentiles for time to stand and to run 10 m in young children. J. Pediatr..

[30] Kuwabara C., Shiba Y., Sakamoto M., Sato H. (2013). The Relationship between the Movement Patterns of Rising from a Supine Position to an Erect Stance and Physical Functions in Healthy Children. Adv. Phys. Educ..

[31] Bohannon R.W., Lusardi M.M. (2004). Getting up from the floor. Determinants and techniques among healthy older adults. Physiother. Theory Pract..

[32] James E.G. (2012). Body movement instructions facilitate synergy level motor learning, retention and transfer. Neurosci. Lett..

[33] Santos De Oliveira I., Da D., Oliveira S., Cattuzzo M.T. (2016). The Effect of Different Instructions in a General Motor Competence and Perceived Competence of Children. J. Phys. Educ. Sport. Manag..

[34] Beenakker E.A.C., Maurits N.M., Fock J.M., Brouwer O.F., van der Hoeven J.H. (2005). Functional ability and muscle force in healthy children and ambulant Duchenne muscular dystrophy patients. Eur. J. Paediatr. Neurol..

[35] Naugle K.M., Higgins T.J., Manini T.M. (2012). Obesity and use of compensatory strategies to perform common daily activities in pre-clinically disabled older adults. Arch. Gerontol. Geriatr..

[36] Manckoundia P., Buatois S., Gueguen R., Perret-Guillaume C., Laurain M.C., Pfitzenmeyer P., Benetos A. (2008). Clinical determinants of failure in balance tests in elderly subjects. Arch. Gerontol. Geriatr..

[37] Klima D.W., Anderson C., Samrah D., Patel D., Chui K., Newton R. (2016). Standing from the floor in community-dwelling older adults. J. Aging Phys. Act..

[38] Manini T.M., Cook S.B., VanArnam T., Marko M., Ploutz-Snyder L. (2006). Evaluating task modification as an objective measure of functional limitation: Repeatability and comparability. J. Gerontol. Ser. A Biol. Sci. Med. Sci..

[39] King L.A., VanSant A.F. (1995). The effect of solid ankle-foot orthoses on movement patterns used in a supine-to-stand rising task. Phys. Ther..

[40] Belt A.B., Hertel T.A., Mante J.R., Marks T., Rockett V.L., Wade C., Clayton-Krasinski D. (2001). Movement Characteristics of Persons with Prader-Willi Syndrome Rising from Supine. Pediatr. Phys. Ther..

[41] Didier J.P., Mourey F., Brondel L., Marcer I., Milan C., Casillas J.M., Verges B., Winsland J.K.D. (1993). The energetic cost of some daily activities: A comparison in a young and old population. Age Ageing.

[42] Ulbrich J., Raheja A., Alexander N.B. (2000). Body positions used by healthy and frail older adults to rise from the floor. J. Am. Geriatr. Soc..

[43] Schwickert L., Boos R., Klenk J., Bourke A., Becker C., Zijlstra W. (2016). Inertial sensor based analysis of lie-to-stand transfers in younger and older adults. Sensors (Switz.).

[44] Schenkman M., Morey M., Kuchibhatla M. (2000). Spinal flexibility and balance control among community-dwelling adults with and without Parkinson’s disease. J. Gerontol. Ser. A Biol. Sci. Med. Sci..

[45] Henwood T.R., Taaffe D.R. (2005). Improved physical performance in older adults undertaking a short-term programme of high-velocity resistance training. Gerontology.

[46] Manckoundia P., Gerbault N., Mourey F., d’Athis P., Nourdin C., Monin M.P., Camus A., Pfitzenmeyer P. (2007). Multidisciplinary management in geriatric day-hospital is beneficial for elderly fallers: A prospective study of 28 cases. Arch. Gerontol. Geriatr..

[47] Geraldes A.A.R., Albuquerque R.B., Soares R.M., Carvalho J., Farinatti P.T.V. (2008). Association between flexibility of the glenohumeral and hip joints and functional performance in active elderly women. Rev. Bras. Fisioter..

[48] Raso V., Greve J.M.D. (2012). Aerobic or resistance exercise improves performance in activities of daily living in elderly women. Rev. Bras. Med. Esporte.

[49] Manckoundia P., Barthélémy E., Bonnot R., d’Athis P. (2020). Impact of an ambulatory physical activity program on balance and motor abilities of retirees: A prospective study. Int. J. Clin. Pract..

[50] Moffett M.A., Avers D., Bohannon R.W., Shaw K.L., Merlo A.R. (2020). Performance and Clinimetric Properties of the Timed Up From Floor Test Completed by Apparently Healthy Community-Dwelling Older Women. J. Geriatr. Phys. Ther..

[51] Wulf G., McNevin N., Shea C.H. (2001). The automaticity of complex motor skill learning as a function of attentional focus. Q. J. Exp. Psychol. A.

[52] Reid K.F., Fielding R.A. (2012). Skeletal Muscle Power: A Critical Determinant of Physical. Exerc. Sport Sci. Rev..

[53] Stodden D.F., True L.K., Langendorfer S.J., Gao Z. (2013). Associations among selected motor skills and health-related fitness: Indirect evidence for Seefeldt’s proficiency barrier in young adults?. Res. Q. Exerc. Sport.

[54] Utesch T., Bardid F., Strauss B. (2019). The relationship between motor competence and physical fitness from early childhood to early adulthood: A meta-analysis. Sport. Med..

[55] Robinson L.E., Goodway J.D. (2009). Instructional climates in preschool children who are at-risk. Part I: Object-control skill development. Res. Q. Exerc. Sport.

[56] Barnett L.M., Morgan P.J., Van Beurden E., Ball K., Lubans D.R. (2011). A reverse pathway? Actual and perceived skill proficiency and physical activity. Med. Sci. Sports Exerc..

