# Supplementary material for: Assessment in the Supine-To-Stand Task and Functional Health from Youth to Old Age: A Systematic Review
Source: Int J Environ Res Public Health. 2020 Aug 10;17(16):5794. doi: 10.3390/ijerph17165794 (PMC7460168; doi:10.3390/ijerph17165794)
Supplement: Supplementary file 1 [file ijerph-17-05794-s001.pdf]

International Journal of
Environmental Research and Public Health

MDPI

SUPPLEMENTARY MATERIAL

Table S1. Risk of bias within articles included in the review regarding the performance of the supine-to-stand (STS) task. (It is ordered by scoring). The table was originally created by the authors.

-	QUESTIONS *															
**AUTHORS (year)**	**1**	**2**	**3**	**4**	**5**	**6**	**7**	**8**	**9**	**10**	**11**	**12**	**13**	**14**	**15**	Total
Belt et al. (2001) [1]	1	1	1	1	1	1	1	1	NA	1	1	1	1	1	1	14
Bergland & Wyller (2004) [2]	1	1	1	1	1	0	1	1	NA	1	1	1	1	1	1	13
Hofmeyer et al. (2002) [3]	1	1	1	1	0	0	1	1	1	1	1	1	1	1	1	13
Hsue, Chen & Wang (2014)[4]	1	1	1	1	0	1	1	1	NA	1	1	1	1	1	1	13
Klima et al. (2016) [5]	1	1	1	1	0	1	1	1	NA	1	1	1	1	1	1	13
Mewasingh et al. (2004) [6]	1	1	1	1	1	1	1	1	NA	1	1	1	1	1	?	13
Nesbitt et al. (2017) [7]	1	1	1	1	0	1	1	1	NA	1	1	1	1	1	1	13
Schwickert et al. (2016) [8]	1	1	1	1	0	1	1	1	NA	1	1	1	1	1	1	13
Schenkman, Morey & Kuchibhatla (2000)[9]	1	1	1	1	0	1	1	1	NA	1	1	1	1	1	1	13
Manckoundia et al. (2020) [10]	1	1	0	1	0	1	1	1	1	1	1	1	1	1	1	13
Nesbitt et al. (2018) [11]	1	1	1	1	1	1	1	0	NA	1	1	1	1	1	1	13
Mankoundia et al. (2007) [12]	1	1	1	1	0	1	1	1	NA	1	1	1	1	1	1	13
Moffett et al. (2020) [13]	1	1	1	1	0	1	1	1	NA	1	1	1	1	1	1	13
Geraldes et al. (2008) [14]	1	1	1	1	1	1	1	1	NA	1	1	1	0	1	1	13
Manini et al. (2006) [15]	1	1	1	1	0	1	1	1	NA	1	1	1	1	1	1	13
Alexander et al. (1997) [16]	1	1	1	1	0	0	1	1	NA	1	1	1	1	1	1	12
Bergland, Jarnlo & Laake (2002) [17]	1	1	1	1	1	0	1	1	NA	1	1	1	1	1	0	12
Bergland & Laake (2005) [18]	1	1	1	1	1	0	1	1	NA	1	1	1	1	1	0	12
Green & Williams (1992) [19]	1	1	1	0	0	1	1	1	NA	1	1	1	1	1	1	12
Hsue, Wang & Chen (2014) [20]	1	1	1	1	0	1	1	1	NA	1	1	1	1	1	0	12
King & Vansant (1995) [21]	1	1	1	0	0	1	1	1	NA	1	1	1	1	1	1	12
Manckoundia et al. (2008) [22]	1	1	1	1	0	0	1	1	NA	1	1	1	1	1	1	12
Schwickert et al. (2016) [23]	1	1	1	0	0	1	1	1	NA	1	1	1	1	1	1	12
Ulbrich, Raheja & Alexander (2000) [24]	1	1	1	1	0	0	1	1	NA	1	1	1	1	1	1	12
Bohannon & Lusardi (2004) [25]	1	1	1	1	0	1	1	1	NA	1	1	0	1	1	0	11
Duncan et al. (2017) [26]	1	1	1	0	0	1	1	1	NA	1	1	1	1	?	1	11
Henwood & Taaffe (2005) [27]	1	1	1	0	0	1	1	0	1	1	1	0	1	1	1	11
Mewasingh et al. (2002) [28]	1	1	1	1	0	0	1	1	NA	1	1	1	1	1	?	11
Naugle et al. (2012) [29]	1	1	1	1	0	0	1	1	NA	1	1	1	1	0	1	11
Ng et al. (2013) [30]	1	1	1	1	0	1	1	1	NA	1	1	1	0	1	?	11
Raso & Greve (2012) [31]	1	1	1	1	0	1	?	0	1	1	1	?	1	1	1	11
VanSant (1988a) [32]	1	1	1	0	0	1	1	1	NA	1	1	1	1	1	?	11
Didier et al. (1993) [33]	1	1	1	1	0	0	1	1	NA	1	1	1	1	0	0	10
Marsala & VanSant (1998) [34]	1	1	1	0	0	1	1	1	NA	0	1	1	1	1	0	10
VanSant (1988b) [35]	1	1	1	0	0	1	1	1	NA	0	1	1	1	1	?	10
Kuwabara et al. (2013) [36]	1	1	1	1	0	1	0	0	NA	1	1	0	1	1	1	10
Beenakker et al. (2005) [37]	1	1	1	0	0	1	0	0	NA	1	1	1	1	1	0	9
n	37	37	36	28	7	27	34	32	4	35	37	33	35	34	25	
***f* (%)**	100	100	97,3	75,7	18,9	73,0	91,9	86,5	10,8	94,6	100	89,2	94,6	91,9	67,6	

*(1) Was the objective clear? (2) Was the literature review relevant to this topic? (3) Was the design adequate for the research question? (4) Was the sample described in detail? (5) Was there a justification for the sample size? (6) Did the subjects sign the consent form (or their parents/supervisors)? (If not described, assume no) (7) Were the outcome measures reliable? (If not described, assume no) (8) Were the outcome measures valid? (If not described, assume no) (9) Was the intervention described in detail? (10) Were the results reported in terms of statistical significance? (11) Were the methods of analysis adequate? (12) Was the clinical importance relevant? (13) Were the conclusions consistent with the methods and results of the study? (14) Are there implications of research results for the clinical practice? (15) Were the limitations of the study recognized and described by the authors? Scores of items: 0 = does not meet criteria; 1 = satisfies the criteria; ? = not clearly described; NA = not applicable.

References

1 Belt, A.B.; Hertel, T.A.; Mante, J.R.; Marks, T.; Rockett, V.L.; Wade, C.; Clayton-Krasinski, D. Movement Characteristics of Persons with Prader-Willi Syndrome Rising from Supine. *Pediatr. Phys. Ther.* **2001**, *13*, 110–121

2 Bergland, A.; Wyller, T.B. Risk factors for serious fall related injury in elderly women living at home. *Inj. Prev.* **2004**, *10*, 308–313.
3 Hofmeyer, M.R.; Alexander, N.B.; Nyquist, L.V.; Medell, J.L.; Koreishi, A. Floor-rise strategy training in older adults. *J. Am. Geriatr. Soc.* **2002**, *50*, 1702–1706.
4 Hsue, B.J.; Chen, Y.J.; Wang, Y.E. The intra- and inter-rater reliability of component analysis of rise from supine in the children with typical development and developmental delay. *Res. Dev. Disabil.* **2014**, *35*, 162–170.
5 Klima, D.W.; Anderson, C.; Samrah, D.; Patel, D.; Chui, K.; Newton, R. Standing from the floor in community-dwelling older adults. *J. Aging Phys. Act.* **2016**, *24*, 207–213.
6 Mewasingh, L.D.; Sékhara, T.; Pelc, K.; Missa, A.M.; Cheron, G.; Dan, B. Motor strategies in standing up in children with hemiplegia. *Pediatr. Neurol.* **2004**, *30*, 257–261.
7 Nesbitt, D.; Molina, S.L.; Cattuzzo, M.T.; Robinson, L.E.; Phillips, D.; Stodden, D. Assessment of a Supine-to-Stand (STS) Task in Early Childhood: A Measure of Functional Motor Competence. *J. Mot. Learn. Dev.* **2017**, *5*, 252–266.
8 Schwickert, L.; Oberle, C.; Becker, C.; Lindemann, U.; Klenk, J.; Schwenk, M.; Bourke, A.; Zijlstra, W. Model development to study strategies of younger and older adults getting up from the floor. *Aging Clin. Exp. Res.* **2016**, *28*, 277–287.
9 Schenkman, M.; Morey, M.; Kuchibhatla, M. Spinal flexibility and balance control among community-dwelling adults with and without Parkinson's disease. *J. Gerontol. Ser. A Biol. Sci. Med. Sci.* **2000**, *55*, 441–445.
10 Manckoundia, P.; Barthélémy, E.; Bonnot, R.; d'Athis, P. Impact of an ambulatory physical activity program on balance and motor abilities of retirees: A prospective study. *Int. J. Clin. Pract.* **2020**, p. e13474
11 Nesbitt, D.; Molina, S.L.; Sacko, R.; Robinson, L.E.; Brian, A.; Stodden, D. Examining the Feasibility of Supine-to-Stand as a Measure of Functional Motor Competence. *J. Mot. Learn. Dev.* **2018**, *6*, 267–286.
12 Manckoundia, P.; Gerbault, N.; Mourey, F.; d'Athis, P.; Nourdin, C.; Monin, M.P.; Camus, A.; Pfitzenmeyer, P. Multidisciplinary management in geriatric day-hospital is beneficial for elderly fallers: A prospective study of 28 cases. *Arch. Gerontol. Geriatr.* **2007**, *44*, 61–70.
13 Moffett, M.A.; Avers, D.; Bohannon, R.W.; Shaw, K.L.; Merlo, A.R. Performance and Clinimetric Properties of the Timed Up From Floor Test Completed by Apparently Healthy Community-Dwelling Older Women. *J. Geriatr. Phys. Ther.* **2020**, doi:10.1519/JPT.0000000000000264.
14 Geraldes, A.A.R.; Albuquerque, R.B.; Soares, R.M.; Carvalho, J.; Farinatti, P.T.V. Association between flexibility of the glenohumeral and hip joints and functional performance in active elderly women. *Rev. Bras. Fisioter.* **2008**, *12*, 274–282.
15 Manini, T.M.; Cook, S.B.; VanArnam, T.; Marko, M.; Ploutz-Snyder, L. Evaluating task modification as an objective measure of functional limitation: Repeatability and comparability. *J. Gerontol. Ser. A Biol. Sci. Med. Sci.* **2006**, *61*, 718–725.
16 Alexander, N.B.; Ulbrich, J.; Raheja, A.; Channer, D. Rising from the floor in older adults. *J. Am. Geriatr. Soc.* **1997**, *45*, 564–569.
17 Bergland, A.; Jarnlo, G.B.; Laake, K. Validity of an index of self-reported walking for balance and falls in elderly women. *Adv. Physiother.* **2002**, *4*, 65–73.
18 Bergland, A.; Laake, K. Concurrent and predictive validity of "getting up from lying on the floor." *Aging Clin. Exp. Res.* **2005**, *17*, 181–185.
19 Green, L.N.; Williams, K. Differences in developmental movement patterns used by active versus sedentary middle-aged adults coming from a supine position to erect stance. *Phys. Ther.* **1992**, *8*, 560–568.
20 Hsue, B.J.; Wang, Y.E.; Chen, Y.J. The movement patterns used to rise from a supine position by children with developmental delay and age-related differences in these. *Res. Dev. Disabil.* **2014**, *35*, 2205–2214.
21 King, L.A.; VanSant, A.F. The effect of solid ankle-foot orthoses on movement patterns used in a supine-to-stand rising task. *Phys. Ther.* **1995**, *75*, 952–964.
22 Manckoundia, P.; Buatois, S.; Gueguen, R.; Perret-Guillaume, C.; Laurain, M.C.; Pfitzenmeyer, P.; Benetos, A. Clinical determinants of failure in balance tests in elderly subjects. *Arch. Gerontol. Geriatr.* **2008**, *47*, 217–228.
23 Schwickert, L.; Boos, R.; Klenk, J.; Bourke, A.; Becker, C.; Zijlstra, W. Inertial sensor based analysis of lie-to-stand transfers in younger and older adults. *Sensors (Switz.)* **2016**, *16*, 1277.

24 Ulbrich, J.; Raheja, A.; Alexander, N.B. Body positions used by healthy and frail older adults to rise from the floor. *J. Am. Geriatr. Soc.* **2000**, *48*, 1626–1632.
25 Bohannon, R.W.; Lusardi, M.M. Getting up from the floor. Determinants and techniques among healthy older adults. *Physiother. Theory Pract.* **2004**, *20*, 233–241.
26 Duncan, M.J.; Lawson, C.; Walker, L.J.; Stodden, D.; Eyre, E.L.J. The Utility of the Supine-to-Stand Test as a Measure of Functional Motor Competence in Children Aged 5-9 Years. *Sports* **2017**, *5*, 1–8.
27 Henwood, T.R.; Taaffe, D.R. Improved physical performance in older adults undertaking a short-term programme of high-velocity resistance training. *Gerontology* **2005**, *51*, 108–115.
28 Mewasingh, L.D.; Demil, A.; Christiaens, F.J.C.; Missa, A.M.; Cheron, G.; Dan, B. Motor strategies in standing up in leukomalacic spastic diplegia. *Brain Dev.* **2002**, *24*, 291–295.
29 Naugle, K.M.; Higgins, T.J.; Manini, T.M. Obesity and use of compensatory strategies to perform common daily activities in pre-clinically disabled older adults. *Arch. Gerontol. Geriatr.* **2012**, *54*, e134–e138.
30 Ng, J.; Conaway, M.R.; Rigby, A.S.; Priestman, A.; Baxter, P.S. Methods of standing from supine and percentiles for time to stand and to run 10 meters in young children. *J. Pediatr.* **2013**, *162*, 552–556, doi:10.1016/j.jpeds.2012.08.030.
31 Raso, V.; Greve, J.M.D. Aerobic or resistance exercise improves performance in activities of daily living in elderly women. *Rev. Bras. Med. do Esporte* **2012**, *18*, 87–90.
32 VanSant, A.F. Age differences in movement patterns used by children to rise from a supine position to erect stance. *Phys. Ther.* **1988a**, *68*, 1330–1338.
33 Didier, J.P.; Mourey, F.; Brondel, L.; Marcer, I.; Milan, C.; Casillas, J.M.; Verges, B.; Winsland, J.K.D. The energetic cost of some daily activities: A comparison in a young and old population. *Age Ageing* **1993**, *22*, 90–96.
34 Marsala, G.; VanSant, A.F. Age-related differences in movement patterns used by toddlers to rise from a suppine position to erect stance. *Phys. Ther.* **1998**, *78*, 149–159.
35 VanSant, A.F. Rising from a supine position to erect stance. Description of adult movement and a developmental hypothesis. *Phys. Ther.* **1988b**, *62*, 185–192.
36 Kuwabara, C.; Shiba, Y.; Sakamoto, M.; Sato, H. The Relationship between the Movement Patterns of Rising from a Supine Position to an Erect Stance and Physical Functions in Healthy Children. *Adv. Phys. Educ.* **2013**, *3*, 92–97.
37 Beenakker, E.A.C.; Maurits, N.M.; Fock, J.M.; Brouwer, O.F.; van der Hoeven, J.H. Functional ability and muscle force in healthy children and ambulant Duchenne muscular dystrophy patients. *Eur. J. Paediatr. Neurol.* **2005**, *9*, 387–393.

© 2020 by the authors. Licensee MDPI, Basel, Switzerland. This article is an open a distributed under the terms and conditions of the Creative Commons Attributi license (http://creativecommons.org/licenses/by/4.0/).
